# Guest Support for Outdoor Smoke-Free Policies within a Homeless Shelter

**DOI:** 10.3390/ijerph19042408

**Published:** 2022-02-19

**Authors:** Jayda Martinez, Midhat Z. Jafry, Tzuan A. Chen, Michael S. Businelle, Darla E. Kendzor, Maggie Britton, Maya Vijayaraghavan, Lorraine R. Reitzel

**Affiliations:** 1 Department of Psychological, Health, & Learning Sciences, College of Education, University of Houston, 3657 Cullen Blvd Stephen Power Farish Hall, Houston, TX 77204, USA; jamart58@cougarnet.uh.edu (J.M.); mzjafry@cougarnet.uh.edu (M.Z.J.); tchen3@central.uh.edu (T.A.C.); mkbritto@central.uh.edu (M.B.); 2 Department of Biology and Biochemistry, College of Natural Sciences & Mathematics, University of Houston, Science & Research Building 2, 3455 Cullen Blvd #342, Houston, TX 77004, USA; 3 HEALTH Research Institute, University of Houston, 4349 Martin Luther King Boulevard, Houston, TX 77204, USA; michael-businelle@ouhsc.edu; 4 TSET Health Promotion Research Center, University of Oklahoma Health Sciences Center, 655 Research Parkway, Suite 400, Oklahoma City, OK 73104, USA; darla-kendzor@ouhsc.edu; 5 Division of General Internal Medicine, University of California, San Francisco, 1001 Potrero Avenue, San Francisco, CA 94110, USA; maya.vijayaraghavan@ucsf.edu

**Keywords:** homelessness, smoking cessation, tobacco control policy, secondhand smoke, environmental tobacco smoke exposure, partial smoking ban

## Abstract

Roughly 70–80% of adults experiencing homelessness smoke cigarettes. Smoke-free living/workplace policies are an empirically-supported tobacco control intervention. However, homeless shelters may be reluctant to implement smoke-free policies due to fears of it discouraging current/potential shelter guests from taking refuge there. The current study was meant to characterize guest support for on-property smoke-free policies within a homeless shelter with an extant indoor tobacco use ban amongst never smokers, former smokers, and current smokers to provide data on this point. Participants comprised a convenience sample of adult guests of a homeless shelter in Texas (N = 394, 28.2% women; 10.2% former; and 75.9% current smokers). Participant sociodemographics, smoking status, behavioral health diagnoses, and support for two versions of an on-property outdoor courtyard smoke-free policy (one partial, one complete) were assessed. Data were collected in two waves in a repeated cross-sectional design. Overall, 64.0% of participants supported a partial, and 32.0% a full smoking ban. Logistic regressions, controlling for wave of data collection, age, sex, and any additional significant predictors from a semi-adjusted model, examined associations between participant characteristics and policy support. Older participants (OR = 1.024, CI_0.95_ = 1.005–1.044), non-veterans (OR = 2.523, CI_0.95_ = 1.156–5.506), former smokers (OR = 2.730, CI_0.95_ = 1.191–6.258), and those without severe mental illness (OR = 1.731, CI_0.95_ = 1.061–2.824) had significantly greater odds of supporting a partial smoking ban. Relative to current smokers, never smokers (OR = 3.902, CI_0.95_ = 2.133–7.137) and former smokers (OR = 8.257, CI_0.95_ = 3.951–17.258) had significantly greater odds of supporting a complete smoking ban. The implementation of smoke-free living/workplace policies in homeless shelters may enjoy more support from guests—specifically, non-smokers—than anticipated by shelter administrators. Aside from reducing ambient smoke exposure for never and former smokers, these policies can help to reduce ubiquitous smoking cues for those who may want to quit, are undergoing a quit attempt, or are trying to maintain abstinence. Interventionists might partner with shelter guests, particularly smokers, to inform the roll-out of such policies for maximal acceptance and adoption.

## 1. Introduction

According to the National Alliance to End Homelessness, homelessness in the United States (US) has increased by 2% between the years 2019 and 2020, resulting in a shift from an estimated 567,715 to 580,466 people experiencing homelessness annually [1]. Moreover, homelessness rates may have accelerated during the adjustment to the COVID-19 economic crises [1]. Approximately 70–80% of adults experiencing homelessness smoke cigarettes [2], a rate five times greater than that found amongst domiciled adults. Smoking is socially and culturally accepted among the community of adults experiencing homelessness [3]; this contributes to unsuccessful quit attempts [4]. Moreover, the various and voluminous stressors encountered by individuals experiencing homelessness can also negatively impact their tobacco use and perceived ability to successfully quit, including food insufficiency [5], unmet medical or surgical care [6], perceived discrimination [7], victimization, exposure to violence [8], and high rates of behavioral health problems and comorbid substance use [9]. Furthermore, high rates of smoking amongst this vulnerable group have been associated with exceedingly high rates of chronic tobacco-related diseases such as cancers, cardiovascular diseases, and heart disease, as well as premature mortality [10,11]. Consequently, it is critical to intervene to mitigate these tobacco-related health inequities amongst homeless adults and to prevent additional morbidity and mortality.

Approximately 49% of adults experiencing homelessness in the US seek sanctuary at homeless shelters [1]. The literature supports that the implementation of tobacco or smoke-free workplace/living policies, whereby tobacco use is prohibited indoors and outdoors, is an effective tobacco control measure [12]. These policies not only lead to an increase in quit attempts and abstinence amongst smokers [13], they also reduce non-smokers’ exposure to environmental tobacco smoke [14] and reduce smoking cues that can engender cravings in former smokers [15]. Although workplace restrictions and laws commonly prohibit tobacco product usage indoors [16], many homeless shelters do not have comprehensive tobacco-free outdoor policies whereby all tobacco use is forbidden on the grounds, instead opting for designated smoking areas, other partial tobacco-free policies (e.g., designated non-smoking areas, time controlled partial outdoor smoking bans) or no outdoor smoking policies at all [17,18,19]. Although shelter administrators likely recognize the detrimental effects of smoking on their stakeholders’ health and health-related quality of life, concerns regarding implementing a comprehensive tobacco-free workplace/living policy include that it will discourage current and potential guests from residing therein and may facilitate negative interactions with the surrounding community by forcing guests to leave the property to smoke [20], making them more vulnerable to victimization and other negative events [8,21]. Additionally, studies suggest that the need to enforce such policies at homeless shelters may deter their implementation [17,18]. Specifically, shelters are a place of last resort for many of these low-income adults [22]; therefore, addressing policy violators with fines or eviction—as may be common in other settings—is inconsistent with their supportive (and, potentially, housing-first) missions, vision, and values [17]. However, only a few studies have addressed what support there may be for tobacco-free workplace/living policies amongst shelter guest/residents themselves [19,20,23,24].

One of the few prior US studies on guests’ support of tobacco-free workplace/living policies was conducted in a homeless shelter in San Diego, CA, that disallowed smoking inside and outside the premises except during four scheduled smoking “breaks,” which were allowed within designated smoking areas further removed from the premises [23]. Investigators interviewed 170 guests (>70% smokers) and reported that three-quarters of the current smokers attributed their reduced cigarette consumption to the shelter’s policy, and around half reported that the policies spurred a quit attempt. Interestingly, most smokers who quit smoking while residing at the shelter thought they could help others quit and were willing to do so formally as “cessation counselors” [23]. Moreover, less than 10% of smoking guests indicated concerns about having to stay at the shelter given their smoking ban [23]. This same research group used qualitative research methods within seven shelter locations in San Diego, CA, USA, with varying smoking policies and found strong support amongst current and former smokers for an indoor and partial outdoor smoking ban, with a majority of those interviewed believing that such policies were associated with reduced tobacco consumption amongst shelter guests [19]. Taken together, these studies suggest rather broad acceptability of smoking bans in this sheltered living environment, and—potentially—support the effect of those policies on smoking cessation and engendering smoking cessation advocacy amongst some guests.

Another notable US study was conducted in the largest homeless shelter for adults in Dallas, Texas [20]. That study collected data on guests’ support for a partial versus a full smoking ban on the shelter grounds, as tobacco use was already disallowed within buildings on the premises. Specifically, a partial policy would have limited smoking/tobacco use to one half of a protected (i.e., only shelter guests can access this space) outside courtyard within the grounds of the shelter. Conversely, a full smoking ban would have disallowed smoking or tobacco use within the open-air courtyard completely. Data were collected from guests prior to any announcement of policy changes and again after the partial smoking ban was implemented. In this case, shelter guests were not active participants in the policy rollout; however, smoking cessation services were available on site before the policy change was envisioned and remained available thereafter. Results indicated that most guests supported the partial ban, both before and after its implementation (60% of *n* = 155 pre-policy; 70% of *n* = 150 post-policy) [19]. There was less support, however, for a full ban (29% of *n* = 155 pre-policy; 36% of *n* = 150 post-policy) [20]. Over half of the sample believed that partial or full bans would have positive impacts on guests’ health, and between 27.4% (pre-ban) and 42.9% (post-ban) of the sample thought it would reduce the smoking rate amongst guests [20]. For the small sample of guests who completed both data collection waves (*n* = 89), the expired carbon monoxide levels of current smokers decreased following the partial smoking ban [20]. Moreover, the study found that there was no significant difference in time spent off the shelter property from before to after the partial ban [20], suggesting that the policy implementation did not change guests’ behaviors in this regard. This study also compared smokers and nonsmokers, within waves of data collection, and found that nonsmokers were more supportive of the full ban at each wave, and more supportive of the partial ban at the second wave of data collection, relative to smokers [20]. However, this study did not fully characterize the differences between guests who did and did not support these policies—overall or by smoking status. Moreover, policy support between groups of non-smokers (i.e., never smokers and former smokers) was not previously assessed.

Given the paucity of literature in this area, the purpose of the current study was to expand upon the extant literature on guest support for smoke-free policies at a homeless shelter through a secondary data analysis of the aforementioned study in Dallas, Texas [20], by (1) pooling data from *unique* participants assessed at either wave of data collection—something not done in the original analysis [20]; (2) further examining characteristics that differentiated those who were in support of a partial and a full smoke-free policy, respectively, for the on-property outdoor courtyard; and (3) comparing policy support between never smokers, former smokers, and current smokers. Understanding more about guests who support these policies may help guide shelter administrators’ decision-making and inform policy roll-out procedures, including the targeting and content of educational and cessation initiatives that can address policy opposition without alienating guests from safe refuge.

## 2. Materials and Methods

### 2.1. Participants

Participants were recruited through flyers posted at a homeless shelter in Dallas, Texas, advertising a study focused on better understanding the health and health-related behaviors of shelter guests. Data collection was accomplished in the summer of 2013, during two data collection periods (i.e., waves) in a repeated cross-sectional design. Shelter guests, who were all at least 18 years of age, were eligible for participation if they were English-speaking and literate at the ≥7th grade level. All study procedures, including screening, informed consent processes, and data collection, were conducted on site. The survey was administered on a computer or tablet in a semi-private area of the shelter (in the administrative office area) with the items read aloud to the participant via headphones. Remuneration for participation was a USD 20 department store gift card. The study was approved by the Institutional Review Boards of The University of Texas Health Science Center and the University of Houston. Informed consent was obtained from all participants. More information regarding the procedures associated with this study is available elsewhere [20].

### 2.2. Measures

#### 2.2.1. Participant Characteristics

Participant characteristics included age, sex (male or female), education (<high school, high school/GED, or >high school), last month’s income from all sources (no income or had income), employment status (at least part time employment or not employed), health insurance status (any type health insurance or no health insurance), veteran status (no or yes), lifetime length of homelessness (in months), number of homeless episodes over the lifetime, the length of time they had been guests of the shelter where the study was conducted (in weeks), the average number of hours per day spent on site at the shelter where the study was conducted, the average number of smokers participants were around on weekdays and weekends, the number of close friends the participant had who smoked cigarettes, whether the participant themselves endorsed regular use of a tobacco product other than conventional cigarettes (no or yes), lifetime history of diagnosis with an alcohol or non-nicotine drug use disorder (no or yes), and lifetime history of diagnosis with a severe mental illness (i.e., major depression, bipolar disorder, and/or schizophrenia/schizoaffective disorder; no or yes).

Participants current smoking status was largely ascertained based on responses to the first two items administered in the survey that queried lifetime cigarette consumption exceeding 100 cigarettes and if they currently smoked. Current smokers were those endorsing that they smoked >100 cigarettes over the lifetime and currently smoked cigarettes. Former smokers were those who endorsed that they had smoked >100 cigarettes over the lifetime but that they did not currently smoke cigarettes. Never smokers were defined as those who reported “no” to having smoked >100 cigarettes over the lifetime. There were 11 participants who could not be classified using these conventions. In three cases, participants endorsed having smoked >100 cigarettes over the lifetime and said they did not currently smoke cigarettes; however, they provided contradictory information by later saying “yes” to a survey item reading “I currently smoke cigarettes.” These participants were classified as current smokers. In three cases, participants skipped each of the initial items but said “yes” to the later survey item reading “I currently smoke cigarettes.” These participants were classified as current smokers. In five cases, participants reported “no” to having smoked >100 cigarettes over the lifetime and were not asked the initial question about their current smoking status due to skip patterning, but later said “no” to the survey item reading “I currently smoke cigarettes.” These participants were coded as never smokers.

#### 2.2.2. Smoking-Related Characteristics

Smoking-related characteristics were only collected from those self-identifying as current smokers. These included the age they started smoking (age of smoking initiation), the average number of cigarettes smoked per day, the number of years smoked, the number of smoking quit attempts across the lifetime, whether they were a daily or intermittent smoker (no or yes), and the time until the first cigarette of the morning (within 5 min of waking, 6–30 min of waking, 31–60 min of waking, or >60 min after waking).

#### 2.2.3. Outdoor Courtyard Smoking Ban Items

Guest support for two versions of an on-property outdoor courtyard smoke-free policy (one partial, one complete) were assessed with the following items: (1) “I support the creation of a smoke free zone in half of the [shelter name] courtyard,” and (2) “I support a complete smoking ban at the [shelter name].” Response options ranged from strongly disagree to strongly agree and were collapsed into strongly agree or agree versus neutral, disagree, or strongly disagree. This convention mirrors that from the prior work [20]. The shelter setting already disallowed all tobacco use indoors and provided on-site tobacco cessation counseling for guests who wanted to make a quit attempt.

### 2.3. Data Analysis

Descriptive statistics of participant’s characteristics (e.g., sociodemographics, severe mental illness, and/or non-nicotine substance use disorder diagnosis), smoking-related characteristics, and attitude toward smoking bans were calculated. Differences between current smokers, former smokers, and never smokers on participant characteristics were examined using analysis of variance or chi-square tests/Fisher’s exact tests for continuous and categorical variables, respectively.

First, semi-adjusted logistic regressions, controlling for wave of data collection, examined the association of participant characteristics and support for smoke-free shelter policies. Then, fully-adjusted logistic regressions adjusting for wave, age, sex, and any additional significant predictors from the semi-adjusted logistic regression analyses were examined. This was considered a full significant-predictors adjusted model.

Next, generalized linear mixed model analyses with binomial distribution and a log link function were conducted on the small sample of guests who completed both data collection waves to examine the interaction between significant predictors and the data collection wave variable to assess whether the associations changed over time. The sample for these analyses was *n* = 88, as one respondent did not provide information on their support of partial/full smoking ban at wave 2.

Lastly, semi-adjusted logistic regression analyses were conducted on a subset of the sample, current smokers only, to assess the associations of current smoker’s smoking-related characteristics and support of the partial and full smoking ban. For the subsample of participants who completed both waves of data collection [20], data from wave 1 were used for analysis.

Odds ratios and the associated 95% confidence levels were reported. All analyses were conducted using SAS version 9.4. Alpha was set at 0.05 [25].

## 3. Results

Among the 394 homeless participants included in this study, 75.9% (*n* = 299) were current smokers, 10.2% (*n* = 40) were former smokers, and 14.0% (*n* = 55) were never smokers. Overall, 28.2% (*n* = 111) were women. Participants were, on average, 43.3 years of age (SD = 11.8), and were homeless for an average of 39.1 (SD = 49.6) months over their lifetime. Additionally, 76.4% (*n* = 301) were uninsured, 8.1% (*n* = 32) were US veterans, 22.3% (*n* = 88) had a non-nicotine substance use disorder, and 68.3% (*n* = 269) had a history of severe mental illness (Table 1).

Overall, 64.0% (*n* = 252) of participants supported a partial courtyard smoking ban, and 32.0% (*n* = 126) supported the implementation of a full courtyard smoking ban at the shelter. Significant differences between current smokers, former smokers, and non-smokers were found on the number of close friends who smoke (*p* = 0.02), regular use of other tobacco products (*p* < 0.01), support of the partial smoking ban (*p* < 0.01), and support of the full smoking ban (*p* < 0.01). Relative to former smokers, current smokers were more likely to have more close friends who smoked (3.6 vs. 1.6) and were more likely to use other (non-conventional cigarette) tobacco products (23.6%% vs. 5.0%). Current smokers were less supportive of a partial smoking ban relative to former smokers and never smokers (59.5% vs. 80.0% vs. 76.4%). The same pattern was found regarding support for a full smoking ban (22.7% vs. 70.0% vs. 54.6%; Table 1).

Of the 299 current smokers, participants reported they started smoking at the age of 17.9 years (SD = 7.1), had smoked for an average of 19.1 years (SD = 11.9), and had made an intentional smoking quit attempt lasting at least 24 h approximately 5.1 (SD = 3.1) times (Table 2).

### 3.1. Support of Partial Outdoor Courtyard Smoking Ban at the Shelter

When adjusted for data collection wave only, results indicated that older (OR = 1.021, CI_0.95_: 1.003–1.039), non-veteran (OR = 2.399, CI_0.95_: 1.144–5.031), former smokers (OR = 2.310, CI_0.95_: 1.014–5.261), and those without severe mental illness (OR = 1.821, CI_0.95_: 1.143–2.901), respectively, each had greater odds of supporting a partial outdoor courtyard smoking ban. Each of these variables retained their statistical significance when entered jointly in a full significant-predictors adjusted model that additionally included data collection wave and sex, (age (OR: 1.024, CI_0.95_: 1.005–1.044), non-veteran (OR: 2.523, CI_0.95_: 1.156–5.506), former smokers (OR: 2.730, CI_0.95_: 1.191–6.258), and those without severe mental illness (OR: 1.731, CI_0.95_: 1.061–2.824)) (Table 3). Finally, models including interactions between data collection wave and age, sex, veteran status, severe mental illness, and smoking status were entered into a generalized linear mixed model predicting the partial ban support amongst the *n* = 88 guests who completed both waves of data collection and provided responses on the dependent variable at wave 2. The results showed that only age was a significant predictor (*p* = 0.046) of partial smoking ban support, but the interaction term of wave and age was insignificant (*p* = 0.787; data not shown). These results suggest that associations between age and partial smoking ban support did not change over time amongst guests completing both waves of data collection.

### 3.2. Support of Full Outdoor Courtyard Smoking Ban at the Shelter

When adjusted for data collection wave only, results showed that being a never smoker (OR = 3.611, CI_0.95_: 1.950–6.688) or former smoker (OR = 8.417, CI_0.95_: 3.854–18.383) was associated with greater odds of supporting a complete outdoor courtyard smoking ban. None of the other factors were significantly associated with the support of a full outdoor smoking ban. This variable maintained statistical significance when entered simultaneously in a single, full significant-predictors model controlling for data collection wave, age, and sex (never smoker (OR: 3.902, CI_0.95_: 2.133–7.137) and former smoker (OR: 8.257, CI_0.95_: 3.951–17.258) (Table 3)). Finally, models including interactions between data collection wave and smoking status revealed that former smoker was a significant predictor (*p* < 0.004) of support for a full outdoor ban, but the interaction term of wave and smoking status was not significant (*p* = 0.548; data not shown). These results suggest that associations between smoking status and full smoking ban support did not change over time amongst guests completing both waves of data collection (*n* = 88).

### 3.3. Support of Partial/Full Smoking Ban among Current Smokers

The results of logistic regression analyses adjusted for data collection wave revealed that none of the smoking-related characteristics (i.e., age of smoking initiation, average cigarettes smoked per day, years smoked, number of lifetime quit attempts, smoking frequency, or time to the first cigarette of the day) were significantly associated with the support of partial or of a full outdoor courtyard smoking ban at the shelter among the current smokers (N = 299; data not shown).

## 4. Discussion

This study was a secondary data analysis that characterized guest support for a partial and a full outdoor courtyard smoking ban, respectively, at a large, adults-only homeless shelter in Dallas, Texas, US. Unlike the original analysis [20], which examined support for smoking bans within discrete waves of data collection, the current work pooled data from both data collection waves—before and after a partial smoking ban was implemented within an interior open-air courtyard on the property—and included wave 1 data from the subset of the sample that completed both data collection waves. Moreover, the present study expanded the assessment of guest characteristics that distinguished those who supported each type of ban (partial and full), including by status as a former smoker, never smoker, or current smoker. This re-analysis was intended to provide a more comprehensive view of shelter guest support for outdoor tobacco-free policies within the context of a real-world overnight shelter setting and to provide additional information to characterize guests who may represent sources of policy support for future tobacco-free policy rollouts in similar settings. There has been a dearth of literature over the last decade on this topic from the perspective of the shelter guest, with most extant work on smoke-free policy implementation in homeless shelters being confined to ~2015 [19,20,23,24] publication or e-publication dates. Coupled with a concomitant tobacco control/intervention research-to-practice translational gap in shelter settings that remains evident in works published more recently (2020–2021) [17,26,27], and in the context of the general “failing” state of tobacco control in Texas more broadly [28], additional literature further characterizing guest support for tobacco-free workplace/living policies and guidance on their implementation seems warranted to further support future change to reduce the implementation gap.

Results of this analysis indicated sizeable support for a partial outdoor courtyard smoking ban: 64% of 394 unique participants overall supported a partial ban. There was less evident support for a full outdoor courtyard smoking ban, with 32% of 394 unique participants in favor. These levels of support largely and necessarily reflect the prior report [20]. However, the unique contribution of this work is in further delineating the nature of that support by guest characteristics to provide information on identifying potential advocates for change that can be harnessed as part of a policy implementation process. To that end, results revealed that former smokers were 2.73 times more likely than current smokers to support a partial tobacco-free ban and 8.26 times more likely than current smokers to support a full smoking ban. Moreover, never smokers were also 3.90 times more likely than current smokers to support a full smoking ban. Thus, results suggest that non-smoking guests may be major sources of support for outdoor tobacco-free shelter policy implementation. Conversely, results suggest the importance of engaging smoking guests prior to any tobacco-free policy implementation to inform the roll-out of such policies for maximal acceptance and adoption. Results also indicated that older guests, those who did not previously serve in the military, and those without a history of serious mental health disorder diagnosis were approximately 1.02 to 2.52 times more likely to support a partial smoking ban at the shelter. Consequently, although more research is needed in other shelter settings to establish generalizability of results, younger guests, those with a military service record, and those with serious mental illness should also be engaged in the rollout of partial smoking bans within shelter settings to enhance understanding, acceptance, and adherence to such policies; these guests can identify barriers to policy implementation or adherence that are especially salient for them that can be proactively addressed. Educational programming regarding the policy change and the benefits of cessation might also be aptly directed toward, with content adapted for, these guests (e.g., programming for those with mental illness can cover mental health benefits of quitting tobacco use, programming for veterans can highlight tobacco marketing practices directed toward this group and the social justice ramifications of these external behavioral manipulation efforts).

Overall, results support that the implementation of smoke-free living/workplace policies in homeless shelters may enjoy more support from guests—specifically former and never smokers—than may be anticipated by shelter administrators who have heretofore been reluctant to implement these tobacco control measures for fear of negative ramifications on guests [17,18]. The results are similar to other work that also suggests the acceptability of smoke-free policies amongst shelter guests (e.g., [19,23]). Aside from reducing ambient smoke exposure for non-smokers [29], these policies can help to reduce ubiquitous smoking cues for those who may want to quit, are undergoing a quit attempt, or who want to maintain abstinence [24,30]. Although the present study cannot verify this, the very strong support from former smokers for partial and full smoking bans may reflect the struggle inherent in maintaining abstinence in a setting rife with smoking cues (e.g., [24]). This is particularly poignant given that respondents were primarily daily smokers, consuming an average of 12 cigarettes per day, and reported a very high exposure to other smokers (around 42–43 smokers a day, on average, and around 48 per day as reported by former smokers), while residing at the shelter. This, paired with data suggesting that the implementation of a partial outdoor smoking ban did not significantly impact time spent off the shelter grounds by its guests [20], supports that shelter administrators should strongly consider the implementation of smoke- or tobacco-free policies for the health and wellness of their guests. This is especially important as studies support adults experiencing homelessness suffer significant tobacco-related health disparities that dramatically reduce their life expectancies [11,31] and because tobacco use amongst this subpopulation is largely untreated by health professionals [4,29]. Thus, there is a critical need for all professionals—not limited to healthcare professionals but including administrators of agencies providing shelter and services to this vulnerable group—to take steps toward the implementation of evidence-based policies and practices to reduce the devastating toll of tobacco use on its stakeholders.

Although former and never smoking guests supported these policies at a higher rate than smoking guests, it is important to acknowledge that non-smokers are a minority in homeless shelters. Thus, shelter directors’ potential ambivalence about evidence-based tobacco policy implementation may reflect the tension between how they can meet the needs of the majority group, smokers with high levels of nicotine dependence, and the minority group of non-smoking clientele who may experience harmful secondhand smoke exposure. To best serve both populations, the importance of making medications and counseling for tobacco use cessation available and easily accessible in the context of policy implementation is paramount. The role of evidence-based medications, such as over-the-counter nicotine replacement therapies, can be twofold: (1) they can mitigate withdrawal symptoms to assist smokers with adherence to the policy, even if they are not interested in smoking cessation; and (2) they can prime cessation behaviors in the context of a smoke-free policy for those who may be interested in smoking cessation [29]. Thus, recommendations to accompany policy changes include: (1) efforts to add or expand cessation services that are available on site, if possible—both for those ready to quit and those who are not yet willing to do so; (2) implementation processes that incorporate buy-in from residents and staff; (3) evident leadership support; (4) troubleshooting guides for staff who will be expected to monitor policy adherence; and (5) violation policies that are not punitive and do not take away shelter [30]. Shelter administrators should explore resources that may be available from local or national tobacco control efforts to assist them in policy implementation and tobacco cessation service provision, including those that may be available from Quitlines, which often may provide counseling and a starter kit of nicotine replacement therapies to users ready to quit. As many shelters are located in urban areas, contacting local universities who train students to be counselors and place them in community settings as part of their education, or who have tobacco research programs, may yield resources for their residents. Many national groups, such as the American Lung Association, offer webinars about cessation treatment for professionals as well as passive dissemination materials promoting cessation, and may be helpful in directing shelter administrators to ongoing sources of funding for cessation services and medications.

Although a comprehensive/full tobacco-free policy that bans tobacco use in all indoor and outdoor spaces is the gold standard in tobacco control [32,33,34], results suggest that a partial outdoor smoking ban may be the most acceptable policy—at least initially—for implementation in shelter settings. Partial bans should include that any outdoor space within shelter grounds has designated and accessible smoke/tobacco-free areas that minimize smoking exposures and cues. For maximal effectiveness, a partial outdoor smoking ban should at the very least ensure that there is no tobacco use and ambient smoke/vape exposure in proximity to entrances and exits of the shelter grounds for nonsmokers and guests trying to cut down or quit tobacco use. Moreover, and especially given the high prevalence of concurrent tobacco product use amongst smokers experiencing homelessness [35,36,37], policies should make mention of all tobacco products, including smokeless tobacco and e-cigarettes. Ensuring the inclusion of all tobacco products in policies can help to prevent the use of an alternative form of tobacco to address cravings, which can hamper intended health promotion efforts [32,35]. Clear signage regarding tobacco-free policies is recommended, and an enforcement plan is advised [35]. In order to align with the values of homeless-serving agencies, policy enforcement might consist of providing policy violators with: (1) brief, non-confrontative education on the policy and the rationale behind it; (2) an invitation from a staff member to accompany the guest to an approved tobacco use area; and/or (3) information about how they can get help to quit or reduce tobacco use (e.g., on-site resources or a referral to a tobacco Quitline).

There is a plethora of resources available to guide the implementation of tobacco-free policies in organizational or residential settings (e.g., public housing complexes), e.g., [36,37]. Generally, this guidance suggests the importance of clear communication with guest stakeholders about the policy, setting a designated policy implementation date that offers time for resident preparation, conducting townhalls or other meetings whereby guests can voice their thoughts regarding the impending changes, and making available interventions to address tobacco use dependence [38]. This guidance has clear applicability to homeless shelter settings and should be considered in implementation planning. Additionally, best practices in implementation science and community-participatory research each suggest the importance of engaging the target community in residential policy changes regarding tobacco control [39,40]. Therefore, it is recommended that shelter leadership seek both smoking and non-smoking “champions” amongst shelter guests to develop grass-roots support for policy implementation efforts. The current work suggests stakeholders, including former smokers, who might be ideal for these efforts. At least one prior study suggests that shelter guests who successfully quit are willing to play an active role in helping their peers quit as well [23,41]; such enthusiasm should be reinforced by shelter administrators and coupled with basic cessation education (e.g., evidence-based cessation methods) and tools (e.g., Quitline information, application-based smoking cessation support for smartphone installation, local support groups). These efforts should supplement agency-based education about the harms of tobacco use and the rationale behind the policy rollout that is delivered to employee and guest stakeholders alike. Due to the potentially transient nature of shelter guests, education and cessation services should be conducted/promoted regularly over time to reach new guests. Finally, those least likely to support tobacco/smoke-free policies should be specifically engaged in implementation planning. Although the current findings require replication in other settings, results suggest that younger guests, military veterans, and adults with a history of serious behavioral health diagnoses are the least likely to support partial outdoor smoking bans, suggesting the utility of their purposeful engagement in informing the rollout and subsequent enforcement.

The limitations of this work include those previously identified [20], including its cross-sectional nature and because it was limited to a single shelter in the south with an extant indoor tobacco use ban and where tobacco cessation services were available on site. Therefore, generalizability of our findings to other settings and samples awaits future study. However, reported data represented ~75% of shelter residents at the time of data collection [20] with a similar sex and race distribution to Dallas’ overall homeless adult population [42]. Other limitations include those inherent in self-reported data. Additionally, participant’s beliefs, opinions, and attitudes may have been influenced, for example, by extant policies (or the lack thereof) in place at the time of assessment. Therefore, beliefs, opinions, and attitudes might have differed if a full outdoor courtyard smoking ban had been implemented, if the shelter did not have an indoor tobacco use ban, if cessation services were not available onsite, or if different strategies were used in the policy rollout, etc. An additional limitation is the age of the dataset used in this analysis (2013); however, substantive changes in smoking prevalence and policy implementation in shelter settings since that time are not widely evident [20,23,27], and Texas has made no substantive progress in tobacco control overall per the latest “grades” assigned by the American Lung Association [28]. Finally, the potential impact of this work is merely incremental; this was a secondary data analysis that offers a complementary picture of previously published work [20] through an alternative data analysis. However, given the current paucity of the literature on characterizing guest support for tobacco/smoke-free shelter policies, even incremental work such as this is needed to guide practice. Although limitations certainly should be considered when interpreting these data, the need to better address tobacco use and dependence of shelter guests with evidence-based policies and practices is both clear and clearly overdue.

## 5. Conclusions

Individuals experiencing homelessness use tobacco at exceedingly high rates and, accordingly, suffer high rates of associated tobacco-related morbidities and premature mortality [10,11]. Tobacco use disorder is often overlooked in healthcare encounters with adults experiencing homelessness [4,17], and effective tobacco control strategies such as comprehensive tobacco-free workplace/living that are not often implemented in shelter settings [19,20]. Although shelter administrators may be hesitant to implement such policies for fear of guest resistance or enforcement issues [17,18], the results of the current study add to a growing literature on the potential acceptability of implementing tobacco/smoke-free living policies. Specifically, the results of this study indicated that ~60–80% of participating guests supported a partial ban and 23–70% supported a full smoke-free ban in the outdoor courtyard, with variance based on smoking status. Nevertheless, it is important to acknowledge that even most current smokers supported at least a partial smoking ban, supporting consideration of policy implementation by shelter directors. Understanding more about guests who are likely to support partial (former smokers, older guests, those without a military background, and those without a history of serious mental illness) or full (former and never smokers) smoking ban in a shelter setting can provide insight into planning an effective administration-led rollout by engaging champions for change from the community itself who can formally or informally generate grass-roots support for the implementation. Moreover, this information can inform the targeting and content of educational and cessation initiatives that can address (and potentially alter attitudes about) tobacco-free policy opposition without alienating shelter guests from safe refuge.

## Figures and Tables

**Table 1:** Participant Characteristics Overall and By Smoking Status (N = 394 adults experiencing homelessness).

	All	Never Smoker	Former Smoker	Current Smoker	Statistic	*p*-Value
	*n* = 394	*n* = 55	*n* = 40	*n* = 299		
Participant Characteristics	Mean (SD) or % (*n*)					
Age	43.3 (11.8)	46.0 (12.6)	42.3 (12.3)	43.0 (11.6)	1.65	0.19
Sex					4.13	0.13
Male	71.8 (283)	61.8 (34)	80.0 (32)	72.6 (217)		
Female	28.2 (111)	38.2 (21)	20.0 (8)	27.4 (82)		
Education					6.54	0.16
<High School	25.4 (100)	14.6 (8)	22.5 (9)	27.8 (83)		
High School/GED	44.2 (174)	45.5 (25)	40.0 (16)	44.5 (133)		
>High School	30.5 (120)	40.0 (22)	37.5 (15)	27.8 (83)		
Last Month’s Income					0.99	0.61
No income	44.8 (164)	51.0 (26)	41.7 (15)	44.1 (123)		
Had Income	55.2 (202)	49.0 (25)	58.3 (21)	55.9 (156)		
Employment Status					0.37	0.83
At least part time employment	9.9 (39)	9.1 (5)	7.5 (3)	10.4 (31)		
Not employed	90.1 (355)	90.9 (50)	92.5 (37)	89.6 (268)		
Health Insurance					1.14	0.57
Any Type Health Insurance	23.6 (93)	21.8 (12)	17.5 (7)	24.7 (74)		
No Health Insurance	76.4 (301)	78.2 (43)	82.5 (33)	75.3 (225)		
Veteran Status					6.09	0.04
No	91.9 (362)	98.2 (54)	97.5 (39)	90.0 (269)		
Yes	8.1 (32)	1.8 (1)	2.5 (1)	10.0 (30)		
Length of Homeless (in months, over lifetime)	39.1 (49.6)	39.9 (49.3)	44.9 (66.2)	38.2 (47.2)	0.32	0.73
# Discrete Episodes of Homelessness (over lifetime)	2.9 (3.0)	3.0 (3.4)	3.5 (4.0)	2.9 (2.7)	0.76	0.47
Length of Time Housed at Shelter (in weeks)	38.7 (55.8)	39.6 (57.6)	39.9 (55.0)	38.4 (55.7)	0.02	0.98
Average Hours per Day Spent in the Shelter	13.3 (5.6)	13.3 (5.7)	12.8 (6.5)	13.4 (5.5)	0.17	0.85
# of Smokers Around (average weekday)	43.3 (38.9)	44.2 (41.1)	48.7 (41.3)	42.5 (38.2)	0.46	0.63
# of Smokers Around (average weekend)	42.2 (39.2)	46.3 (42.3)	48.4 (41.7)	40.7 (38.2)	1.03	0.36
# of Close Friends who Smoke ^‡^	3.3 (4.1)	3.2 (5.4)	1.6 (1.7)	3.6 (3.9)	4.13	0.02 ^‡^
Other Tobacco Product Use					21.25	<0.01
No	78.6 (264)	100.0 (50)	95.0 (38)	76.4 (226)		
Yes	21.4 (72)	0 (0)	5.0 (2)	23.6 (70)		
Substance Use Disorder (by history)					3.45	0.18
No	77.7 (306)	87.3 (48)	77.5 (31)	75.9 (227)		
Yes	22.3 (88)	12.7 (7)	22.5 (9)	24.1 (72)		
Severe Mental Illness (by history)					5.65	0.06
No	31.7 (125)	45.5 (25)	27.5 (11)	29.8 (89)		
Yes	68.3 (269)	54.6 (30)	72.5 (29)	70.2 (210)		
**Outdoor Courtyard Smoking Ban Items**	**% (*n*)**				**Statistic**	***p*-Value**
Partial Smoking Ban					10.68	<0.01
Strongly Disagree, Disagree, Neutral	36.0 (142)	23.6 (13)	20.0 (8)	40.5 (121)		
Agree, Strongly Agree	64.0 (252)	76.4 (42)	80.0 (32)	59.5 (178)		
Full Smoking Ban					51.19	<0.01
Strongly Disagree, Disagree, Neutral	68.0 (268)	45.5 (25)	30.0 (12)	77.3 (231)		
Agree, Strongly Agree	32.0 (126)	54.6 (30)	70.0 (28)	22.7 (68)		

Note. ^‡^ Significant difference was found between former smokers and current smokers (*p* < 0.01).

**Table 2:** Smoking-Related Characteristics (N = 299 adult smokers experiencing homelessness).

Smokers’ Characteristics	Mean (SD)/% (*n*)
Age of Smoking Initiation	17.9 (7.1)
Average Cigarettes Smoked Per Day	12.0 (7.2)
Years Smoked	19.1 (11.9)
# Quit Attempts (lifetime)	5.1 (3.1)
Smoking Frequency	
Sometimes	18.1 (53)
Everyday	81.9 (240)
Time to 1st Cigarette of the Day	
Within 5 min of waking	35.5 (104)
6 to 30 min of waking	33.4 (98)
31 to 60 min of waking	13.3 (39)
>60 min after waking	17.7 (52)

**Table 3:** Results of Semi- and Fully-Adjusted Logistic Regression Analyses on the Association of Participant Characteristics and Support for Partial and Full Outdoor Courtyard Smoking Bans in the Shelter Setting (N = 394 adults experiencing homelessness).

	Support of Partial Smoking Ban				Support of Full Smoking Ban			
	Semi Adjusted Models		Full Adjusted Model		Semi Adjusted Models		Full Adjusted Model	
Participant Characteristics	OR	CI_0.95_	OR	CI_0.95_	OR	CI_0.95_	OR	CI_0.95_
Age	1.021	(1.003, 1.039)	1.024	(1.005, 1.044)	1.016	(0.997, 1.035)	1.016	(0.996, 1.037)
Male	1.157	(0.733, 1.825)	1.062	(0.650, 1.734)	1.017	(0.634, 1.632)	0.978	(0.585, 1.635)
High school/GED (ref: < High school)	1.241	(0.722, 2.134)			0.637	(0.369, 1.099)		
>High school (ref: < High school)	1.246	(0.694, 2.237)			0.655	(0.363, 1.184)		
Had Income in Last Month	1.104	(0.718, 1.696)			1.391	(0.891, 2.172)		
At Least Part Time Employed	1.213	(0.599, 2.456)			0.979	(0.477, 2.011)		
Had health Insurance	1.382	(0.836, 2.286)			1.064	(0.647, 1.749)		
Non-veteran	2.399	(1.144, 5.031)	2.523	(1.156, 5.506)	1.102	(0.502, 2.418)		
Length of Homeless (in months, over lifetime)	1.002	(0.997, 1.006)			1.003	(0.999, 1.007)		
# Discrete Episodes of Homelessness (over lifetime)	0.975	(0.911, 1.044)			1.000	(0.932, 1.074)		
Length of Time Housed at Shelter (in weeks)	1.002	(0.998, 1.006)			1.003	(0.999, 1.007)		
Average Hours per Day Spent in the Shelter	0.981	(0.945, 1.018)			0.999	(0.962, 1.038)		
# of Smokers Around (average weekday)	0.997	(0.992, 1.002)			1.001	(0.995, 1.006)		
# of Smokers Around (average weekend)	0.998	(0.993, 1.003)			1.001	(0.996, 1.007)		
# of Close Friends who Smoke	1.018	(0.963, 1.075)			0.963	(0.908, 1.022)		
No Substance Use Disorder by history	1.539	(0.944, 2.508)			0.967	(0.582, 1.607)		
No Severe Mental Illness by history	1.821	(1.143, 2.901)	1.731	(1.061, 2.824)	1.183	(0.753, 1.858)		
Never Smoker (ref: Current smoker)	1.915	(0.975, 3.762)	1.805	(0.907, 3.592)	3.611	(1.950, 6.688)	3.902	(2.133, 7.137)
Former Smoker (ref: Current smoker)	2.310	(1.014, 5.261)	2.730	(1.191, 6.258)	8.417	(3.854, 18.383)	8.257	(3.951, 17.258)

Note. OR: Odds Ratio; CI: Confidence Interval; Semi-adjusted models adjusted for wave of data collection; full significant-predictor adjusted models adjusted for wave of data collection, age, and sex, while including any additional significant variables from the semi-adjusted models.

## Data Availability

The data presented in this study are available on request from the corresponding author. The data are not publicly available due to ethical restrictions based on in-formed consent agreements.
